# The Ca^2+^ Sensor STIM in Human Diseases

**DOI:** 10.3390/biom13091284

**Published:** 2023-08-22

**Authors:** Alejandro Berna-Erro, Jose Sanchez-Collado, Joel Nieto-Felipe, Alvaro Macias-Diaz, Pedro C. Redondo, Tarik Smani, Jose J. Lopez, Isaac Jardin, Juan A. Rosado

**Affiliations:** 1 Department of Physiology, Institute of Molecular Pathology Biomarkers, Universidad de Extremadura, 10003 Caceres, Spain; alejandrobe@unex.es (A.B.-E.); joelnf@unex.es (J.N.-F.); alvaromd@unex.es (A.M.-D.); pcr@unex.es (P.C.R.); jjlopez@unex.es (J.J.L.); 2 Department of Medical Physiology and Biophysics, University of Seville, 41004 Seville, Spain; josesc@unex.es (J.S.-C.); tasmani@us.es (T.S.); 3 Group of Cardiovascular Pathophysiology, Institute of Biomedicine of Seville, University Hospital of Virgen del Rocio, University of Seville, Spanish National Research Council (CSIC), 41004 Seville, Spain

**Keywords:** STIM1, STIM2, store-operated Ca^2+^ entry, Orai

## Abstract

The STIM family of proteins plays a crucial role in a plethora of cellular functions through the regulation of store-operated Ca^2+^ entry (SOCE) and, thus, intracellular calcium homeostasis. The two members of the mammalian STIM family, STIM1 and STIM2, are transmembrane proteins that act as Ca^2+^ sensors in the endoplasmic reticulum (ER) and, upon Ca^2+^ store discharge, interact with and activate the Orai/CRACs in the plasma membrane. Dysregulation of Ca^2+^ signaling leads to the pathogenesis of a variety of human diseases, including neurodegenerative disorders, cardiovascular diseases, cancer, and immune disorders. Therefore, understanding the mechanisms underlying Ca^2+^ signaling pathways is crucial for developing therapeutic strategies targeting these diseases. This review focuses on several rare conditions associated with STIM1 mutations that lead to either gain- or loss-of-function, characterized by myopathy, hematological and immunological disorders, among others, and due to abnormal activation of CRACs. In addition, we summarize the current evidence concerning STIM2 allele duplication and deletion associated with language, intellectual, and developmental delay, recurrent pulmonary infections, microcephaly, facial dimorphism, limb anomalies, hypogonadism, and congenital heart defects.

## 1. Introduction

Calcium (Ca^2+^) plays a vital role as a second messenger in the animal kingdom. Ca^2+^ intervenes in a wide range of cellular processes, including cell signaling, gene expression, fertilization, muscle contraction, and neurotransmitter release. The precise regulation of intracellular Ca^2+^ levels is essential for maintaining cellular homeostasis and proper behavior of various physiological processes. Ca^2+^ acts as a versatile signaling molecule due to its ability to rapidly change its cytosolic concentration in response to extracellular stimuli. This dynamic regulation requires the synergistic action of various Ca^2+^ sensors, ion channels, pumps, and transporters that control Ca^2+^ efflux and influx across intracellular compartments and the plasma membrane (PM) [1]. Among the mechanisms involved in the maintenance of intracellular calcium homeostasis, the store-operated Ca^2+^ entry (SOCE) is the most relevant in non-excitable cells. By facilitating the influx of Ca^2+^ from the extracellular medium to the cytoplasm in response to the depletion of intracellular Ca^2+^ stores, mainly the endoplasmic reticulum (ER), SOCE plays a crucial role in various physiological and pathological conditions [2,3,4,5].

The identification of SOCE as a distinct mechanism for calcium entry was a significant breakthrough in the field of cell physiology. In the late 1970s, some researchers observed that certain cells exhibited sustained calcium influx following depletion of intracellular calcium stores. This phenomenon was termed “capacitative” or “store-operated” calcium entry, suggesting that it was dependent on the depletion of intracellular stores rather than extracellular factors [6]. The first candidates proposed to mediate SOCE were called as “calcium-release-activated channels” (CRACs) [7]. These channels were hypothesized to be responsible for the sustained influx of calcium ions following store depletion. However, identifying and characterizing these channels proved to be a challenging task due to their elusive nature and lack of specific markers (for more information, see [8]. Furthermore, numerous models attempted to elucidate the mechanism underlying the communication and activation of CRACs in the PM upon ER depletion. Nevertheless, a comprehensive understanding of this process remained yet unclear [9,10,11].

During the last two decades, substantial advancements have been achieved in clarifying the complex and well-orchestrated molecular mechanisms that control SOCE. The identification of two pivotal family proteins, stromal interaction molecule (STIM) [12,13] and Orai [14,15], revolutionized our understanding of this process. The two members of the STIM family, STIM1 and STIM2, are ER-resident proteins that act as a sensor for intraluminal calcium levels [Ca^2+^]_ER_. Upon store depletion, STIM proteins undergo conformational changes and translocate to specialized regions called puncta or ER-PM junctions [16]. On the other hand, the three members of the Orai family, Orai1, Orai2, and Orai3, exhibit similar structure, a four transmembrane-spanning PM protein with the N- and C-terminal regions facing the cytoplasm (for more information, see [17]. The three Orai proteins possess the ability to form highly selective calcium channels that are triggered by STIM proteins upon store depletion and are the constituents of the CRACs [18,19]. The interaction between STIM and Orai proteins is crucial for the activation of SOCE and subsequent calcium entry (for more information, see [20]). The discovery of STIM1 and Orai1 provided the missing pieces of the puzzle and established a molecular basis for understanding the mechanism of SOCE [21].

Understanding the molecular basis of SOCE has not only shed light on fundamental cellular processes but also has important implications for human health. Dysregulation of the pattern of expression and/or function of SOCE main components, STIM and Orai proteins, have been implicated in various diseases, including immune disorders, muscular and neurodegenerative diseases, cardiovascular conditions, and cancer. Therefore, elucidating the mechanisms underlying SOCE and its associated proteins holds great promise for developing novel therapeutic strategies targeting these diseases. In the following sections, we aim to provide a comprehensive review of the implications of STIM proteins in the development and progression of various diseases.

### The STIM Family

**Figure 1:**
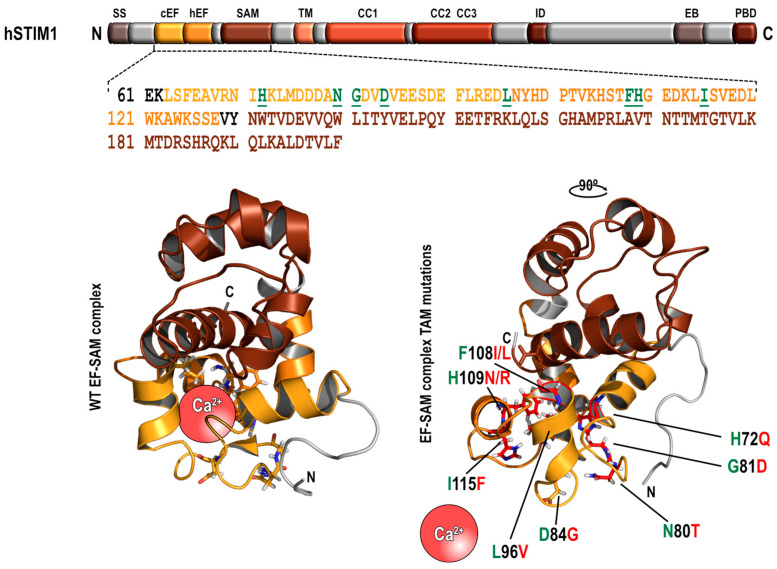
TAM-associated point mutations. Top: Cartoon of full-length STIM1 showing the overall structure with important regions highlighted and amino acid sequence of the EF-SAM region. Bottom: Models of the EF-SAM complex with the amino acids susceptible to TAM mutations highlighted. SS, signal sequence; cEF, “canonical” EF-hand domain; hEF, “hidden” EF-hand domain; SAM, sterile alpha motif; TM, transmembrane domain; CC, coiled-coil domains; ID, inhibitory domain; EB, EB1-binding domain; PBD, polybasic domain.

STIM1 was first identified as a type I transmembrane protein with an undefined role but relevant in the pathogenesis of rhabdomyosarcomas and rhabdoid tumors [22]. Nowadays, it is well-known that the two members of the STIM family are dimeric, one single transmembrane-spanning protein primarily located in the ER membrane. However, a subpopulation of the proteins may also be found in acidic stores or to a lesser extent in the PM [12,13,22,23,24,25]. Both, STIM1 and STIM2, exhibit the N-terminal domain within the ER lumen (amino acids (aa) 1–213 STIM1, aa 1–217 STIM2), a single transmembrane domain (aa 214–234 STIM1, aa 218–238 STIM2), with a larger C-terminal portion located in the cytosol (aa 235–685 STIM1, aa 323–721 STIM2) (Figure 1). In addition to functioning as remarkably proficient sensors of luminal Ca^2+^ in the ER, STIM proteins possess structural regions that allow them to form homomers and interact with and regulate Ca^2+^-selective channels in the PM [12,13,14,15,16,26].

STIM1 and STIM2 can detect changes in [Ca^2+^]_ER_, which is in the range of 100 to 800 μM. The initial studies by nuclear magnetic resonance revealed that the N-terminal portion of STIM proteins contains paired EF-hand structures that form a hydrophobic pocket, which is stabilized by the sterile α-motif (SAM) domain (aa 131–200 STIM1, aa 136–204 STIM2) and results in a compact globular structure. Whereas the canonical EF-hand domain (aa 63–96 STIM1, aa 67–100 STIM2) binds Ca^2+^, with the half-maximum activation occurring at 200 μM (STIM1) and 400 μM (STIM2), the second non-canonical or hidden EF-hand motif (aa 97–128 STIM1, aa 101–132 STIM2) stabilizes the canonical EF-hand, supported by the action of the SAM domain [27,28,29,30]. The STIM1 EF-SAM complex is tightly folded and contains a hydrophobic pocket, where Ca^2+^ binds, which encompasses 11 residues (V68, I71, H72, L74, M75, L92, L96, K104, F108, I115, and L120) of the EF-hand. This pocket is secured by L195 and L199 of the SAM domain of STIM1. The arrangement of negatively charged residues on the surface of this globular motif includes D77, D78, D82, D84, E86, E87, D89, E90, E94, and D95 from the canonical EF-hand; E111, D112, E118, and E128 from the non-canonical EF-hand; and D135, E136, and D196 from the SAM domain. These negatively charged residues likely prevent intermolecular interactions with other N-terminal STIM1 domains [27,28,29,30,31].

The luminal and cytosolic domains of STIM proteins are divided by a single transmembrane helix that spans the ER membrane (aa 215–234 STIM1, aa 218–238 STIM2) [31]. This TM domain contributes to the resting state of STIM1, as mutations in residues I220W and C227W resulted in constitutive STIM1 activation. It was proposed that, within the dimer and in the resting state, the C-terminal segments of the two TM domains interact (aa 221–232), whereas the N-terminal segments do not associate, forming a crossing angle of approximately 45° (aa 212–220). Upon STIM activation, the N-terminal sections come closer together facilitating STIM conformational change from folded to elongated [32]. However, this model has been recently challenged by new data obtained by single-molecule Förster resonance energy transfer (FRET) analysis of STIM1 C-terminus dimer that shows that the TM domains are separated in the resting state [33].

The C-terminus of STIM proteins is characterized by three coiled-coil (CC) domains, namely, CC1, CC2, and CC3 (aa 236–437 STIM1, aa 243–431 STIM2). These CC domains are vital for the interaction with and gating of Orai proteins, and therefore, SOCE. The function of the CC domains was independently identified almost simultaneously by three groups, who named it as the Orai-activating small fragment (OASF), composed of CC1, CC2, and CC3 [19], CRAC-activating domain (CAD) [34], and the STIM-Orai-activating region (SOAR) [18], both located in CC2 and CC3. The CC1 domain comprises three α-helices (α1, α2, and α3), while CAD/SOAR is composed of four α-helices (Sα1, Sα2, Sα3, and Sα4) [35,36,37,38,39,40]. As detailed in Section 2, the STIM1 R304W gain-of-function mutation, within the CC1α2 domain, results in constitutive oligomerization and activation of STIM1 proteins, which turn into pathological conditions [41]. Following the CC domains, the C-terminus of STIM proteins contains additional domains responsible for regulation (inhibitory domain; aa 470–491 STIM1, aa 479–487 STIM2) [42] and microtubule end binding (EB; aa 642–645 STIM1) [43,44]. At the very end of the protein sequence is a lysine-rich polybasic domain (PBD), which facilitates interaction with negatively charged phospholipids in the PM [18]. For more information about STIM proteins, see the elegant and thorough revision by Sallinger [45].

As we discuss in the following sections, point mutations or polymorphisms in any of the mentioned motifs lead to STIM1 abnormal function gain/loss, hence, altered SOCE, and subsequently, pathophysiological conditions.

## 2. Disorders Associated with Gain-of-Function STIM1 Mutations

As mentioned above, STIM1 is involved in a plethora of cellular functions and a number of disorders have been associated with STIM1 mutations that lead to either gain- or loss-of-function resulting in constitutive activation of CRACs or deficient Ca^2+^ influx upon depletion of the intracellular Ca^2+^ stores. Among the most relevant disorders associated with gain-of-function mutations of STIM1 are Stormorken syndrome, York platelet syndrome (YPS), and tubular aggregate myopathy (TAM).

### 2.1. Stormorken Syndrome and Tubular Aggregate Myopathy (TAM)

The Stormorken syndrome is a rare autosomal-dominant genetic condition identified by Helge Stormorken in 1984 in a patient suffering from long-lasting bleeding after a minor accident [46]. This syndrome is characterized by bleeding diathesis, thrombocytopenia, congenital miosis, mild anemia, asplenia, headache, ichthyosis, and proximal muscle weakness. One of the most relevant features of this syndrome is the platelet phenotype. While platelets from normal subjects exhibit a very low resting cytosolic free Ca^2+^ concentration, which is significantly elevated upon stimulation with physiological agonists, leading to exposure of phosphatidylserine at the plasma membrane [47,48], Stormorken platelets show significantly elevated resting cytosolic Ca^2+^ concentration and reduced or absent SOCE upon Ca^2+^ store depletion, which has been attributed to near-maximal Orai1 activation at resting conditions [41]. Therefore, platelets from Stormorken patients are in a pre-activated or procoagulant state, leading to prothrombotic predisposition and thrombocytopenia. Consistent with this, non-stimulated platelets from Stormorken patients exhibit increased levels of CD63 and p-selectin at the plasma membrane, as well as alpha granule secretion, which are well-known markers of activation [49]. Paradoxically, it could be expected that constitutive SOCE and procoagulant platelet activity in Stormorken patients might result in an enhanced incidence of thromboembolisms; however, this functional disorder leads to patients with mild bleeding resulting from a reduced platelet cohesion as determined under shear stress conditions both in vivo and ex vivo [41,50]. This phenotype is similar to that exhibited by mice bearing the activating STIM1 EF-hand mutation D84G [51].

Almost three decades after the identification of this condition, the molecular basis of the syndrome was identified as a gain-of-function mutation located in the α2 segment of the STIM1 coiled-coil region 1 (CC1), the STIM1-R304W [41,49,52] (Table 1). The missense mutation occurs in exon 7 of STIM1 (c.910C>T). Expression of the STIM1-R304W mutant in zebrafish resulted in thrombocytopenia, bleeding, and hypoplastic caudal vein, a phenotype that is reminiscent of the Stormorken syndrome in humans [52]. As mentioned above, one of the functions of the STIM1 CC1 domain is to keep STIM1 in a tight and compact state at high ER Ca^2+^ concentrations. This mechanism depends on the formation of a coiled-coil clamp involving the CC1 and CC3 domains [32,53]. Although the precise mechanism has not been fully elucidated, it has been reported that STIM1 carrying the R304W mutation at the end of the CC1α2 segment exhibits CC1 homomerization and elongation of CC1, thus preventing CC1−CC3 clamp formation and maintaining STIM1 in a constitutively active state in the absence of Ca^2+^ store depletion [36]. Consistent with this, NIH3T3 murine fibroblasts transfected with mouse STIM1-R304W fused with yellow fluorescent protein (YFP) showed increased clustering of YFP signal at resting conditions than STIM1-WT, which was evenly distributed in the ER, thus indicating constitutive STIM1 aggregation and subsequent activation [54]. In addition to the STIM1-R304W mutation, the R304Q has also been reported in a smaller number of patients. This mutation causes a milder clinical form of Stormorken syndrome as compared to patients bearing the R304W mutation [55] (Table 1).

In 2021, the genetic analysis of a 12-year-old Chinese female with Stormorken-like symptoms identified a novel heterozygous missense mutation (c.1095G>C transition) leading to a STIM1 K365N amino acid substitution [55] (Table 1). This mutation is located in the CC2 domain and affects directly the SOAR/CAD region involved in the activation of Orai1. This new STIM1 mutation widens the spectrum of STIM1 variants causing Stormorken syndrome.

The Stormorken syndrome clinically overlaps with TAM, a progressive muscle disorder associated with weakness, cramps, myalgia, increased creatine kinase levels, and accumulation of packed membrane tubules containing sarcoplasmic reticulum proteins. These tubules are observed by electron microscopy as straight, single- or double-walled tubules that are highly ordered and aligned in parallel in a longitudinal muscle section [56,57]. Furthermore, TAM is characterized by large variations in fiber size, increased number of fibers with internalized nuclei, as well as type 1 fiber predominance [58]. Most patients with Stormorken syndrome exhibit TAM. Furthermore, TAM is present in a non-syndromic form of TAM as well as in the Stormorken-like syndrome, which lacks miosis and hematological disorders [59]. The most typical clinical manifestation is proximal muscle weakness in lower limbs followed by extraocular muscle weakness [60].

TAM is mediated by several autosomal dominant mutations in the EF-hand domain of STIM1, which result in constitutive CRAC activation. Initially, four activating STIM1 mutations were identified through Sanger sequencing of the STIM1 coding exons and exon–intron boundaries in families of patients with common histological features or by exome sequencing of TAM patients: H72Q, D84G, H109N, and H109R (Figure 1; Table 1). Expression of all the STIM1 mutants in C2C12 myoblasts resulted in constitutive STIM1 oligomerization and clustering, thus demonstrating constitutive activation of STIM1 [60]. Further studies from the same group identified four novel STIM1 EF-hand mutations in six new TAM families: N80T, L96V, F108I, and F108L (Figure 1; Table 1). As for the above-mentioned STIM1 EF-hand mutations, the additional mutations were shown to induce STIM1 clustering in C2C12 myoblasts independently of changes in the ER Ca^2+^ concentration [61]. Two more STIM1-activating mutations were identified in TAM patients, the STIM1-I115F [62] and STIM1-G81D [63]. In contrast to the other TAM STIM1 mutations, myoblasts from patients bearing the G81D mutation did not show an abnormally increased basal cytosolic Ca^2+^ concentration but the amplitude of SOCE was significantly higher in TAM vs. control myoblasts [63].

TAM might also be caused by mutations in other proteins, including Orai1 and the reticular Ca^2+^-handling protein calsequestrin-1. Three missense mutations in the calsequestrin-1 gene have been identified in patients with a mild phenotype associated with tubular aggregate myopathy, including D44N, G103D, and I385T [64]. Furthermore, the Orai1 mutation P245L located in the fourth transmembrane domain has been associated with a Stormorken-like syndrome showing congenital miosis and TAM but lacking hematological abnormalities [52].

### 2.2. York Platelet Syndrome (YPS)

The York platelet syndrome was first reported in a series of articles published in 2003 describing ultrastructural abnormalities found in platelets from a woman and her male child who manifested life-long thrombocytopenia although functionally normal in aggregation [65,66,67,68]. The patients exhibit slightly enlarged platelets with the presence of two giant organelles that originate in megakaryocytes and fully develop in circulating platelets, the opaque organelles, large electron opaque bodies, and the target organelles made up of multiple layers resembling a target [69]. Analytical electron microscopy revealed that these aberrant organelles contain large amounts of Ca^2+^ and phosphorous, in a ratio that resembles that observed in normal platelet dense bodies, as well as peroxidase and acid phosphatase activity, like primary lysosomes. As the number of lysosomes was reduced in YPS platelets, it has been reported that both opaque and target organelles are either abnormal lysosomes or fused with lysosomes during their development [67]. Some platelets contain a normal number of alpha granules, while others present a fewer number or lack this organelle [69]. In addition to the mentioned platelet features, the YPS patients exhibit myopathy characterized by the presence of degenerating and regenerating fibers and an increased number of myofibers with internalized nuclei [69,70].

Whole exome sequencing from YPS patients revealed two *STIM1* variants, c.343A>T and c.910C>T, encoding for the STIM1 mutations I115F and R304W, respectively [69,70] (Table 1). These two mutations have also been recognized as activating STIM1 mutations in TAM and Stormorken syndrome, respectively (see above). In fact, YPS patients have been reported to present tubular aggregates, which is not surprising given the genotypic overlap with TAM and Stormorken syndrome [71].

A recent study [72] has reported five novel STIM1 mutations not related to TAM, Stormorken syndrome, or YPS but leading to muscle phenotype in individuals between 26 and 57 years old. The reported *STIM1* variants c.312A>T, c.412G>A, c.1889C>T, c.2246G>A, and c.1894_1897del encode for the STIM1 mutations K104N, located in the non-canonical EF-hand region, V138I, located in the SAM domain, S630F (in the longer STIM1 isoform (STIM1L) sequence [73]) and R749H (STIM1L), located in C-terminal regions not associated with functional domains, as well as a single frameshift variant, H632fs (STIM1L), lacking the cytosolic proline/serine-rich domain and the polybasic C-terminal region, respectively. Patients bearing these mutations manifested muscle disorders. The patient with K104N showed tubular aggregates, the V138I was associated with congenial fiber type size disproportion, S630F and R749H were associated with type I fiber atrophy and severe muscle dystrophy with inflammatory infiltrates, respectively, and the H632fs mutation was associated with mild variation in fiber size [72]. Functional analysis of SOCE in HEK293 cells expressing STIM1 WT or the STIM1 mutants K104N, V138I, S630F, and R749H has revealed that all the mutations enhance SOCE, especially the V138I and S630F mutations [72]. Furthermore, all these mutations have been reported to enhance significantly the resting cytosolic Ca^2+^ concentration when expressed in HEK293 cells [74], thus suggesting that these novel STIM1 mutants are gain-of-function variants. There is no further functional analysis of the STIM1-H632fs mutant.

The aberrant SOCE mediated by the aforementioned gain-of-function STIM1 mutations, as well as some gain-of-function Orai1 variants, with the exception of the R304Q, has been recently reported to be sensitive to two structurally unrelated SOCE modulators, CIC-37, a compound developed from a class of pyrazole-bearing molecule, and CIC-39, a biphenyl-triazole developed from Synta66 [74]. If these compounds prove to be tolerable in clinical trials, like other SOCE modulators [75,76], their applicability in the treatment of Stormorken syndrome, TAM, and YPS will be of great relevance.

## 3. Disorders Associated with Loss-of-Function STIM1 Mutations

CRACs are formed by the heterogeneous association of Orai and STIM family members. Twenty years ago, the study of STIM1 and Orai1 loss-of-function mutations allowed the identification of the molecular mediator of SOCE [15]. Indeed, due to their highly similar phenotypic traits, the syndromes arising from STIM1 and Orai1 loss-of-function mutations are grouped under the name of CRAC channelopathy [77]. Although SOCE is a ubiquitous mechanism in human physiology, patients suffering from CRAC channelopathy present a limited spectrum of symptoms that include severe combined immunodeficiency-like disease with chronic infections and autoimmunity, ectodermal dysplasia, abnormal enamel, anhidrosis, mydriasis, and muscular hypotonia [3,59]. This discrepancy reflects the inability of some cells to compensate for the loss of Orai1 or STIM1 function, an issue that undisturbed tissues might solve by using other family members or by adapting an alternative signaling mechanism.

Up to date, six autosomal recessive STIM1 loss-of-function mutations have been described (Table 1). STIM1 E128R (E128fs*9) mutation results from an adenine insertion after position 380 in the *STIM1* gene. Due to the appearance of a premature termination codon, this frameshift produces a truncated STIM1 mutant that ends at the beginning of the SAM domain. The STIM1 E128fs*9 patients’ derived fibroblasts showed markedly reduced STIM1 mRNA levels and an undetectable STIM1 protein expression, making these cells unable to mediate SOCE [78]. Patients suffering from this mutation present a similar phenotype to those with Orai1 deficiency, which is characterized by autoimmune hemolytic anemia and immune thrombocytopenia, hepatosplenomegaly, lymphadenopathy, hypoglycemia, and nonprogressive muscular hypotonia. Byun et al. described another STIM1 null mutation originated by guanine to alanine substitution at the −1 position of STIM1 exon 8 (1538-1G>A) [79]. This mutation was found in a child who died from Kaposi sarcoma at two years of age, a neoplasm caused by human herpesvirus (HHV)-8 infection [80]. In patient-derived EBV-transformed B cells, the STIM1 1538-1G>A substitution induces the formation of abnormally spliced STIM1 mRNA variants and the inability to synthesize functional STIM1 proteins, a condition that abrogates SOCE in these cells [79]. Based on this information, authors claimed that the STIM1 1538-1G>A null mutation may have induced T-cell deficiency in this patient, a condition that favored the opportunistic HHV8 infection and the rapid progression of Kaposi sarcoma.

CRAC channelopathy derived from STIM1 P165Q mutation shows a unique phenotypic trait. This mutation reduced STIM1 expression and function but might still allow for residual SOCE [81]. Although the functional consequences of P165Q mutation are unknown, it has been proposed that this modification impairs STIM1 function by impeding SAM domain homomerization, a key step in the activation mechanism of this protein [30]. Interestingly, the residual Ca^2+^ entry observed in T cells derived from STIM1 P165Q patients might support immune responses, increasing their lifespan compared to other CRAC channelopathy patients. Furthermore, this hypomorphic mutation has been associated with additional inflammatory disorders, such as psoriasis and colitis, that were not previously associated with other STIM1 mutations.

Two loss-of-function mutations have been described within the SOAR/CAD domain of STIM1. As described above, this sequence plays a crucial role in the oligomerization of STIM molecule and the activation of store-operated Ca^2+^ channels. STIM1 R429C mutation was found in two siblings born to consanguineous parents. A younger sister died from sepsis at the age of 21 months, while the older presented dental enamel defect, anhidrosis, generalized eczema, mild muscular hypotonia, light-insensitive pupils, primary enuresis, and combined immunodeficiency. Interestingly, STIM1 R429C mutation did not alter protein expression but impaired SOCE in regulatory and CD3+ T cells [82]. A study on the structural consequences of the STIM1 R429C mutation revealed that this modification destabilizes the CC3 domain, thus inducing the extension of the C-terminal region of the protein and the release of the STIM1 polybasic domain. These alterations promote the constitutive translocation of STIM1 to ER-PM junctions, impair STIM1 oligomerization, and abrogate its ability to activate the Orai1 channel [83]. Wang et al. described a case where the STIM loss-of-function mutation R426C leads to a mild clinical phenotype characterized by enamel defects with rapid dental attrition, nail dysplasia, and frequent throat infections [84]. Like STIM1 R429, R426 is involved in the regulation of STIM1 C-terminus conformational state, a role that assures the stability of this region at resting and following the depletion of reticular Ca^2+^ stores [53]. The STIM1 R426C mutation has been reported to destabilize the CC1-SOAR clamp but also to attenuate moderately the interaction of STIM1 with Orai1, thus reducing the Orai1 currents [85]. The latter provides an explanation for the phenotype reported by Wang et al. [84].

A novel STIM1 loss-of-function mutation was identified in two patients affected with muscle weakness, dysmorphic facies, hypoplastic patellae, dental abnormalities, and hyperelasticity. This phenotype, which only presents mild immune deficiencies, was caused by a guanine insertion after position 685 in the coding sequence of STIM1, a frameshift that changes phenylalanine 229 to leucine and induces a premature termination codon. As observed in other STIM1 null mutations, cells derived from patients carrying STIM1 F229L mutation show reduced STIM1 mRNA levels and an indetectable protein expression [86]. However, the authors did not assay SOCE in patient-derived immune cells, information that might help to clarify the wide phenotypic spectrum evoked by STIM1 loss-of-function mutations.

## 4. STIM2 in Human Diseases

The STIM2 gene is located within the p15.2 band of the short arm of chromosome 4 and adjacent to the p15.1 band (https://www.ensembl.org, accessed on 20 June 2023). There are no more than 40 cases reporting interstitial deletions or duplications on the proximal short arm of chromosome 4 and few involving alterations in STIM2 alleles. The most prevalent phenotype associated with such chromosomal alterations is intellectual disability, presenting other clinical features to a minor degree, such as physical and facial dimorphism or congenital heart diseases [87,88]. For instance, two studies involving STIM2 allele duplication were reported. The first case involves a 22-year-old patient bearing a inherited translocation t(4;8) (p15.2;p23.2) between a region of 33.8 kb (chr4:27358601-27392379, hg19) and 2.9 kb (chr8:4008854-4011844, hg19) [89]. This translocation involved the insertion of a third STIM2 allele into intron 3 of one of the two CSMD1 alleles. Consequently, STIM2 mRNA was found overexpressed in the blood. The proband also carried an additional deletion at 5q12.3 comprising the RGS7BP gene, but the authors were unable to measure both CSMD1 and RGS7BP expression in blood. The father and sister were also bearers of the chromosomal alterations, and all three suffered from migraine and epilepsy. It is difficult to say which gene is the main contributor to the syndrome, because all three are involved in neurological functions [89]. A good candidate would be CSMD1, because its haploinsufficiency has been previously associated with cases of familial epilepsy. However, the authors were unable to measure CSMD1 mRNA expression to identify possible alterations [89]. STIM2 might contribute to the cognitive phenotype since it regulates synaptic plasticity and the stability of mushroom spines in neurons [90]. Regarding that STIM proteins have cellular overlapping functions [91], and that patients bearing the gain-of-function mutation of STIM1 R304W suffer from severe migraines [49], it is tempting to speculate that overexpressed STIM2 might lead to a similar phenotype. The second study identified a 2-year-and-5-month-old proband carrying a duplication of a region of 4.5 Mb in size at 4p15.2p15.1 (chr4: 23,829,778–28,305,958) containing CCKAR, LGI2, RBPJ, and STIM2 genes [92]. Moreover, parental genetic analyses were normal, indicating a “de novo” duplication. The proband displayed language, intellectual, and developmental delay, recurrent pulmonary infections, microcephaly, facial dimorphism, limb anomalies, hypogonadism, and congenital heart defects.

Two studies involving STIM2 allele deletion and haploinsufficiency were reported. The first study performed genetic analyses on a 3-year-old proband displaying physical and intellectual delay, microcephaly, diverse facial anomalies, anxiety, frequent laughing, and hypermobility of the elbows. A deletion of a region of 3.4 Mb in size was found at 4p15.2-p14, generating haploinsufficiency in STIM2 and 15 additional genes [87]. From those 16 genes, the authors restricted to five (DHX15, PCDH7, PPARGC1A, RBPJ, and STIM2) the major contributors to the phenotype. The alteration was inherited from the mother, who also displayed obvious clinical alterations, such as intellectual disability and facial abnormalities. The second report described two 10- and 25-year-old patients bearing a deletion in 4p15.2-p15.3, where STIM2 is located, who showed intellectual and moderate growth delay and obvious physical and facial alterations. For instance, a long and triangular face, deep-set eyes, bilateral ptosis, a prominent lower lip with retrognathia, and an asymmetric chest with kyphoscoliosis to the left [93]. Apart from that, no disease linked to single nucleotide mutations, or specific STIM2 gene deletions, has been described yet. The altered neurological phenotype observed in these patients is consistent with the phenotype observed in STIM2 knockout mice. Mice lacking STIM2 protein showed neurological disorders that led to defective learning abilities [94], which might be an indicator of a defective maturation of the neuronal system.

## 5. Conclusions

STIM proteins, mainly STIM1, play a crucial role in cell biology as a result of the regulation of intracellular Ca^2+^ homeostasis, more precisely, the activation of Ca^2+^ influx through CRACs. Because of the key physiological role of STIM1, several point mutations associated with STIM1 gain-of-function lead to multi-systemic disorders with overlapping features, including platelet dysfunction and thrombocytopenia, bleeding diathesis, or congenital myopathy. Although the Stormorken syndrome, TAM, and YPS are rear conditions with a well-recognized pathogeny associated with mutations in the N-terminal STIM1 region, certain STIM1 polymorphisms lead to diffuse phenotypes not clearly associated with them that deserve further studies. Furthermore, several STIM1 loss-of-function mutations have been identified which lead to moderate or complete impairment of CRAC activation and are associated with immune deficiency, impaired enamel formation, or nail dysplasia, among other disorders. Several disorders have also been associated with STIM2 allele duplication or deletion, leading to STIM2 overexpression or absence, which are characterized by intellectual delay, microcephaly, physical and facial dimorphism, or congenital heart diseases. Characterizing and understanding the genetic and molecular mechanisms underlying STIM protein function are crucial for developing therapeutic strategies targeting these diseases.

## Figures and Tables

**Table 1:** STIM1 gain- and loss-of-function mutations. TAM, tubular aggregate myopathy; YPS, York platelet syndrome.

Point Mutation	Disease	Patient Symptoms
Gain-of-function STIM1 mutations		
STIM1-R304W/Q	Stormorken syndrome	Bleeding diathesis, thrombocytopenia, miosis, mild anemia, asplenia, headache, ichthyosis, and proximal muscle weakness.
STIM1-R304W	YPS	Enlarged platelets with giant organelles called “opaque organelles”, target organelles, and reduced number of alpha granules. Myopathy with tubular aggregates and asplenia/hyposplenia.
STIM1-K365N	Stormorken syndrome	Bleeding diathesis, headache, stroke-like episodes, anemia, thrombocytopenia, and mild myopathy.
STIM1-H72Q	TAM	Muscle weakness, cramps, myalgia, elevated levels of creatine kinase, accumulation of packed membrane tubules highly ordered and aligned in parallel, large variations in muscle fiber size, type 1 fiber predominance, and increased number of fibers with internalized nuclei.
STIM1-N80T	TAM	
STIM1-G81D	TAM	
STIM1-D84G	TAM	
STIM1-L96V	TAM	
STIM1-F108I/L	TAM	
STIM1-H109N/R	TAM	
STIM1-I115F	TAM	
	YPS	Enlarged platelets with giant organelles called “opaque organelles”, target organelles, and reduced number of alpha granules. Myopathy with tubular aggregates and asplenia/hyposplenia.
STIM1-K104N	Muscle disorders	Muscle tubular aggregates.
STIM1-V138L	Muscle disorders	Congenial fiber type size disproportion.
STIM1L-S630F	Muscle disorders	Type I fiber atrophy.
STIM1L-R749H	Muscle disorders	Severe muscle dystrophy with inflammatory infiltrates.
STIM1L-H632fs	Muscle disorders	Mild variation in fiber size.
Loss-of-function STIM1 mutations		
STIM1-E128R	Not classified	Autoimmune hemolytic anemia and immune thrombocytopenia, hepatosplenomegaly, lymphadenopathy, and nonprogressive muscular hypotonia.
STIM1 1538-1G>A	Not classified	T-cell deficiency.
STIM1 P165Q	Not classified	Immune and inflammatory disorders.
STIM1-R429C	Not classified	Dental enamel defect, anhidrosis, generalized eczema, mild muscular hypotonia, light-insensitive pupils, primary enuresis, and combined immunodeficiency.
STIM1-R426C	Not classified	Enamel defects with dental attrition, nail dysplasia, and frequent throat infections.
STIM1-F229L	Not classified	Mild immune deficiency, muscle weakness, dysmorphic facies, hypoplastic patellae, dental abnormalities, and hyperelasticity.

## Data Availability

The data presented in this study are available on request from the corresponding author.
